# Hypothesizing High Negative Emotionality as a Function of Genetic Addiction Risk Severity (GARS) Testing in Alcohol Use Disorder (AUD)

**DOI:** 10.15761/jsin.1000245

**Published:** 2020-11-14

**Authors:** Kenneth Blum, Richard Green, Jessica Smith, Luis Llanos-Gomez, David Baron, Rajendra D Badgaiyan

**Affiliations:** 1 Graduate College, Western University Health Sciences, Pomona, CA, USA; 2 Institute of Psychology, ELTE Eötvös Loránd University, Budapest, Hungary; 3 The Kenneth Blum Neurogenetic & Behavioral Institute (Division of iVitalize Inc.), Austin, TX, USA; 4 Division of precision Medicine, Precision Translational Medicine, San Antonio, Tx, USA; 5 Department of Psychiatry, Icahn School of Medicine Mt Sinai, New York, NY, USA; 6 Department of Psychiatry, South Texas Veteran Health Care System, Audie L. Murphy Memorial VA Hospital, San Antonio, TX, Long School of Medicine, University of Texas Medical Center, San Antonio, TX, USA.

An estimated 14.5 million United States (U.S.) citizens ages 12 and older consume enough alcohol to meet the diagnosis for alcohol use disorder (AUD), as reported by the Center for Behavioral Health Statistics and Quality in 2018. A National Survey on Drug Use and Health (NSDUH) conducted in 2019, revealed that 85.6 percent of adults admit to consuming alcohol at some point during their life, 69.5 percent of adults admit to having consumed alcohol within the past year, and 54.9 percent (51.0 percent of adult females and 59.1 percent of adult males) self-report to have consumed alcohol within the past month. The survey also reported that among adults, 25.8 percent (22.2 percent of adult females and 29.7 percent of adult males) reported engaging in binge drinking within the past 30 days. The survey also revealed the 6.3 percent of adults (4.5 percent of adult females and 8.3 percent of adult males) reported to engage in heavy alcohol use within the past 30 days [1]

While there are several effective treatment options for AUD, attrition, and relapse are common in AUD treatment, especially in terms of the numbers of DWI arrests [2]. It is well-known that 10-35% of subjects drop out from AUD clinical trials, and more than 60% relapse in the year following treatment [3]. While precision medicine including genetic antecedents may provide important information that could help identify specific DNA variants in the Brain Reward Circuitry, it is important to identify negative emotionality, especially in repeat DWI offenders [4].

It is known that AUD-type patients with negative emotionality have a higher likelihood of benefitting from favorable outcomes following mindfulness-based relapse prevention [5]. The utilization of the Alcohol Addiction Research Domain Criteria (AARDoC) has been proposed to more thoroughly identify the underlying factors behind AUD (including genetics). The ultimate goal of AARDoC is to effectively advance the development of new genetic-based precision medicine for the treatment of AUD [6].

Along these lines we hypothesize one potential associative role of the Genetic Addiction Risk Severity (GARS) test is to help identify genetic polymorphic antecedents, especially reward genes involving six neurotransmitter systems, in patients with high emotional negativity and multiple DWIs. Our hypothesis is that high Negative Emotionality is a function of GARS testing in DWI Offenders.

## Brief explanation of AUD:

Alcohol Use Disorder (AUD), colloquially known as alcoholism, manifests its pathology through a three-stage cycle: 1) The user experiences an inability to regulate alcohol consumption (binging, intoxication), 2) The user suffers from withdrawal symptoms in the absence of alcohol following a period of intoxication (negative affect, withdrawal), 3) The user feels an extreme craving for alcohol and proceeds to seek it out and consume it (anticipation/preoccupation) [7].

The nature of the progression through the cycle by individuals may be different; the intensity of each stage and the etiology regarding the underlying neurobiology may also vary. The striatum and nucleus accumbens are the two predominant neuroanatomies responsible for the onset of the intoxication/binging stage. The nucleus accumbens (NAc) is known to mediate the pleasurable experience of drugs and alcohol [8]. Repeated activation of the NAc by alcohol consumption may induce changes in the striatum (a habit-forming structure of the brain) which may manifest in the compulsive seeking of alcohol. Repeated exposure between drinking and specific environmental cues may create cognitive associations within the brain reward neurocircuitry. Eventually, stimuli associated with drinking, such as people, locations, or even internal mood states, can illicit a craving response in the absence of alcohol. This phenomenon of associative learning is referred to as Facilitation of Incentive Salience, [9] and helps to explicate the overwhelming desire and compulsive seeking of alcohol that takes place when alcoholics are exposed to cues they have associated with drinking.

The absence of alcohol leads to a phenomenon known as the negative affect/withdrawal stage. In this stage, the reward neurocircuitry is left in a deficit state stemming from the stress of intoxication which results in higher levels of stress neurotransmitters in the extended amygdala and also a dysregulation in the brain’s ability to employ stress mitigating neurotransmitters [10]. The product of such effects is self-reported feelings of anxiety, irritability, and unease that commonly accompany alcohol withdrawal - as well as all addictive substances. For example, the repeated stress of alcohol intoxication and withdrawal interrupts the normal functioning of the brain reward system through the disruption of neurotransmitters and conversely sensitizes the anti-reward stress neurocircuitry. This is accomplished via epigenetic methylation on the DRD2 gene (and likely others), which results in a reduced ability to experience normal degrees of pleasure in people suffering from AUD. In fact, an observation by Hill & Sharma [11] concluded that methylation in the DRD2 gene was significantly associated with familial high-risk status. Moreover, they reported that significant familial risk group differences were also observed in High-Risk (HR) individuals expressing reduced Left Interior Temporal, Left Insula, and Left Fusiform volume relative to Low-Risk (LR) controls. In AUD, the motivation to consume alcohol arises from malfunctioning reward neurocircuitry, conditioned environmental cue effects, and increased activation within the brain stress system. These components work synergistically to manifest excessive drinking behaviors and are key elements in the onset of sensations that lead to relapse during the anticipation/preoccupation stage of AUD [12]. The region of the brain responsible for executive function (planning and organizing) known as the prefrontal cortex serves a pivotal function in the anticipation/preoccupation stage of AUD [13]. The function of the prefrontal cortex can be parceled into two antagonistic systems: a “go” system that initiates habitual responding and impulsive behavior, and a “no-go” system that moderates inhibition to the “go” responses which exerts control over the brain stress system and regulates impulsive behavior. Excess activity in the “go” system, or insufficient activity in the “no-go system”, may lead to binge drinking, and result in increased stress reactivity and a heightened response to alcohol-associated cues, both of which can enhance cravings for alcohol that trigger a relapse. The adaptations underlying the neurophysiology of AUD can persist long into abstinence and contribute to the perennial nature of this disease of reward and habit forming (the basal ganglia), stress (the extended amygdala), and executive function (the prefrontal cortex).

## Why Alcohol Addiction Research Domain Criteria (AARDoC):

In efforts to more accurately understand the variability within AUD, Litten, and colleagues [5] have proposed the Alcohol Addiction Research Domain Criteria (AARDoC) as a configuration to organize research on the genetic, neurobiological, and behavioral components of AUD. The crux behind the motivation of the AARDoC is to enhance the nature targetted medicine for AUD. Nevertheless, continuing study on individual characteristics - that mediate therapeutic outcomes as well as application of new knowledge from research necessitates a common array of tests that elucidates upon the various manifestations of AUD; such information should be accessible to both clinicians and researchers.

Building from AARDoC, Kwako, *et al*. [6] presented an armature for assessing manifoldness in individuals suffering from AUD, called the Addictions Neuro-clinical Assessment (ANA). The focus of ANA is to assess three functional domains implicated in the etiology of the Reward Deficiency Syndrome (RDS) addiction cycle [14]: incentive salience, executive function, and negative emotionality [15]. Recent observations have substantiated this model with factor analytic techniques. Specifically, a three-factor model, representing negative emotionality, incentive salience, and executive function, with self-report and neuropsychological indicators provided an adequate fit to the reported data among a non-treatment seeking and treatment-seeking sample with and without AUD [6].

## Negative Emotionality:

Self-report measures of aggression, anxiety, positive urgency, neuroticism, extraversion, and agreeableness have categorized negative emotionality as a unidimensional domain. In addition, negative emotionality also refers to the propensity of experiencing higher degrees of negative affect and the perception of the world as being a stressful, problematic, and threatening place [16]. Higher scoring individuals on levels of negative emotionality are more prone to frequent and intense episodes of negative emotion such as anger and anxiety and self-report higher levels of stress even in the absence of negative environmental stimuli [16,17].

Substance use is associated with negative emotionality and associated constructs (e.g., neuroticism) in a range of community and clinical samples. Beyond the effects of alcohol intoxication, negative emotionality is associated with increased incidents of substance use problems [18-20] and alcohol-related harmful behavior [18]. Evidence stemming from a number of longitudinal studies alludes to negative emotionality as a predictor of later substance abuse and dependence [19-27].

The association between substance use and negative affect is most commonly presumed to relate to negative reinforcement. That is, substance use may be promoted by the onset of negative emotions as a way to ameliorate or escape undesirable states [28-32]. However, evidence also supports the existence of an alternative neurobiological mechanism that can be interpreted in terms of positive reinforcement: small emotion -based dysregulation. Such a mechanism may promote individuals to behave in immediate reward-seeking behaviors, such as risky drugs and alcohol use, without regard to - long term negative outcomes [33-37]. That is to say, negative affect can sabotage attempts to stop compulsive behaviors [38].

Heavier drinking at baseline, as well as heavier and more frequent drinking at 6- and 12-month assessments, was associated with negative emotionality. These findings add to previous research by Kwako and colleagues [5,6] by demonstrating that increased negative emotionality was also highly correlated with relapse involved in the regulation of negative affect, moderately correlated to relapse in response to craving and withdrawal, and was not correlated with relapse stemming from social pressure. Altogether, these observations give credence to the construct validity of the negative emotionality domain in people who seek treatment for AUD. Moreover, negative emotionality was found to be highly correlated with drinking to regulate negative affect. In addition, multiple studies have also shown that coping motives are correlated with higher rates of drinking-related problems [39].

Indeed, it is essential to understand negative emotionality in terms of motivation to consume alcoholic beverages and its potential role in drug reinstatement and relapse. Because of the well-known genetic association regarding these two unwanted events, utilizing the GARS test should provide identification of specific genetic antecedents across the brain reward circuitry and even potential epigenetic effects [40-41].
